# Supplementary material for: Modeling glioblastoma heterogeneity as a dynamic network of cell states
Source: Mol Syst Biol. 2021 Sep 16;17(9):e10105. doi: 10.15252/msb.202010105 (PMC8444284; doi:10.15252/msb.202010105)
Supplement: Supplementary file 6 — Source Data for Figure 5 [file MSB-17-e10105-s004.zip › Figure5A_sourcedata/GSEA_3071/nosigdb_stateIII.GseaPreranked.1622795155562/NB_KI_CLUSTER1.html]

Details for gene set NB\_KI\_CLUSTER1[GSEA]

|  || Dataset | state33071 |
| Phenotype | NoPhenotypeAvailable |
| Upregulated in class | na\_pos |
| GeneSet | NB\_KI\_CLUSTER1 |
| Enrichment Score (ES) | 0.9241219 |
| Normalized Enrichment Score (NES) | 5.9408274 |
| Nominal p-value | 0.0 |
| FDR q-value | 0.0 |
| FWER p-Value | 0.0 |
Table: GSEA Results Summary

  

Fig 1: Enrichment plot: NB\_KI\_CLUSTER1      
 Profile of the Running ES Score & Positions of GeneSet Members on the Rank Ordered List

  

| PROBE | GENE SYMBOL | GENE\_TITLE | RANK IN GENE LIST | RANK METRIC SCORE | RUNNING ES | CORE ENRICHMENT || 1 | RPS29 |  |  | 7 | 0.630 | 0.0489 | Yes |
| 2 | RPS12 |  |  | 8 | 0.615 | 0.0983 | Yes |
| 3 | RPS25 |  |  | 19 | 0.527 | 0.1381 | Yes |
| 4 | RPLP1 |  |  | 25 | 0.514 | 0.1781 | Yes |
| 5 | RPL23A |  |  | 27 | 0.502 | 0.2182 | Yes |
| 6 | RPL28 |  |  | 32 | 0.498 | 0.2572 | Yes |
| 7 | RPLP2 |  |  | 36 | 0.489 | 0.2958 | Yes |
| 8 | RPS6 |  |  | 41 | 0.480 | 0.3333 | Yes |
| 9 | RPL13A |  |  | 45 | 0.467 | 0.3701 | Yes |
| 10 | RPL32 |  |  | 55 | 0.457 | 0.4045 | Yes |
| 11 | RPL18A |  |  | 58 | 0.453 | 0.4404 | Yes |
| 12 | RPS18 |  |  | 61 | 0.451 | 0.4762 | Yes |
| 13 | RPL39 |  |  | 72 | 0.442 | 0.5091 | Yes |
| 14 | RPS7 |  |  | 75 | 0.436 | 0.5437 | Yes |
| 15 | RPS3A |  |  | 82 | 0.429 | 0.5766 | Yes |
| 16 | RPS19 |  |  | 84 | 0.427 | 0.6107 | Yes |
| 17 | RPS3 |  |  | 90 | 0.420 | 0.6432 | Yes |
| 18 | RPS27A |  |  | 95 | 0.413 | 0.6754 | Yes |
| 19 | RPL27A |  |  | 104 | 0.408 | 0.7062 | Yes |
| 20 | RPL34 |  |  | 105 | 0.407 | 0.7389 | Yes |
| 21 | RPS27 |  |  | 132 | 0.388 | 0.7634 | Yes |
| 22 | RPL21 |  |  | 141 | 0.382 | 0.7920 | Yes |
| 23 | RPL13 |  |  | 152 | 0.372 | 0.8194 | Yes |
| 24 | RPS2 |  |  | 228 | 0.325 | 0.8263 | Yes |
| 25 | RPS15A |  |  | 245 | 0.312 | 0.8473 | Yes |
| 26 | RPS8 |  |  | 248 | 0.311 | 0.8718 | Yes |
| 27 | RPL41 |  |  | 265 | 0.303 | 0.8920 | Yes |
| 28 | RPL10 |  |  | 285 | 0.296 | 0.9109 | Yes |
| 29 | TPT1 |  |  | 324 | 0.285 | 0.9241 | Yes |
Table: GSEA details [plain text format]

  

Fig 2: NB\_KI\_CLUSTER1: Random ES distribution      
 Gene set null distribution of ES for **NB\_KI\_CLUSTER1**
